# How Do You Get along with Nitrous Oxyd?

**Published:** 1867-08

**Authors:** 


### "HOW DO YOU GET ALONG WITH NITROUS OXYD?"

We have been asked this question so often, both by tongue and pen, that we feel impelled to answer, through the Register, to save time. Here we intend to be very brief, hoping before long to embody our thoughts and experiences in a paper for one of our local societies.

Well—like whisky, nitrous oxyd "is a very good thing in its place." We can only speak favorably of it, after a good deal of experience in its use, and a good deal of research into its nature. We *feel*, more than ever, the vast importance of having an absolutely pure gas; and we take more and more care in its preparation. When *pure* we have confidence in it. As proof of this, we have administered it in cases of epileptic patients, have many times given it where there was organic disease of the heart, given it to patients suffering with phthisis, given it to very old, and quite young patients, to very fat, and to very lean ones, to plethoric, and to spare ones, and expect to continue to do so.

Now, we would very much prefer to have healthy patients; but when a doubtful patient comes for our professional services, the inquiry is, what is best for the patient? not, Can we afford to risk reputation? A sudden shock of severe pain is very dangerous to a patient having serious organic disease of the heart. If the danger is lessened by preventing the pain, the operation being necessary, the pain must be prevented.

Nitrous oxyd is peculiarly adapted to short, severe operations. It is not likely to supercede other anæsthetics in tedious ones. We gave this same opinion at the meeting of the American Dental Convention at Saratoga, a few years ago, and have found no reason to change it yet.

If nitrous oxyd were not good it would be speedily consigned to eternal infamy, through the ignorance and presumption of many who are trying to use it. We could fill a number of the Register with the details of ludicrous items illustrating this point. Not long ago a man called to make arrangement with us to send him, by steamboat, a bottlefull of the gas as he might need it. He could send down by packet, and get his bottle filled and brought back as the boat returned, and he would have the patient wait. And he wanted to know if a quart bottle would be large enough. He was practicing "medicine and dentistry" both, being driven to this course, as he said, by the wants of his community. We have not met any with wilder ideas about it than his, but very many not much tamer. W.

